# Editor's Letter-Box

**Published:** 1899-05-27

**Authors:** 


### Editor's Letter-Box.

[Our Correspondents are reminded that prolixity is a great bar to publication, and that brevity of style and conciseness of statement greatly facilitate early insertion.]

Dr. George Granville Bantock, referring to a recent article in The Hospital entitled "Surgical Cleanliness," writes: "The only remark that calls for notice by me is contained in your last sentence: 'Why do they boil their ligature silk if not to kill the germs it may contain?' Would you be surprised to know that I never boiled a ligature in my life? This is one of the things with which I have been credited by the effort of some too lively imagination."

*** What we said was, "It is fair to ask, as was asked when this paper was read, why do they boil," &c. It was, we believe, Mr. Bowreman Jessett who asked the question.—Ed. *T. H.*

